# Iron Modified Titanate Nanotube Arrays for Photoelectrochemical Removal of *E. coli*

**DOI:** 10.3390/nano11081944

**Published:** 2021-07-28

**Authors:** Chia-Hung Chen, Yen-Ping Peng, Ming-Hsun Lin, Ken-Lin Chang, Yung-Chang Lin, Jian Sun

**Affiliations:** 1 Institute of Environment Engineering, National Sun Yat-sen University, Kaohsiung 804, Taiwan; chiahungchen@g-mail.nsysu.edu.tw (C.-H.C.); klchang@mail.nsysu.edu.tw (K.-L.C.); 2 Department of Marine Environmental Engineering, National Kaohsiung University of Science and Technology, Kaohsiung 811, Taiwan; m9033617@nkust.edu.tw; 3 Center for Environmental Toxin and Emerging-Contaminant Research, Cheng Shiu University, Kaoshiung 804, Taiwan; 4 Department of Electrical Engineering, Cheng Shiu University, Kaoshiung 804, Taiwan; 5 School of Environmental Science and Engineering, Guangdong University of Technology, Guangzhou 510006, China; sunjian472@163.com

**Keywords:** titanate nanotube arrays, antibacterial, *E. coli*, photoelectrochemical

## Abstract

This study used iron modified titanate nanotube arrays (Fe/TNAs) to remove *E. coli* in a photoelectrochemical system. The Fe/TNAs was synthesized by the anodization method and followed by the square wave voltammetry electrochemical deposition (SWVE) method with ferric nitrate as the precursor. Fe/TNAs were characterized by SEM, XRD, XPS, and UV-vis DRS to investigate the surface properties and light absorption. As a result, the iron nanoparticles (NPs) were successfully deposited on the tubular structure of the TNAs, which showed the best light utilization. Moreover, the photoelectrochemical (PEC) properties of the Fe/TNAs were measured by current-light response and electrochemical impedance spectroscopy. The photocurrent of the Fe/TNAs-0.5 (3.5 mA/cm^2^) was higher than TNAs (2.0 mA/cm^2^) and electron lifetime of Fe/TNAs-0.5 (433.3 ms) were also longer than TNAs (290.3 ms). Compared to the photolytic (P), photocatalytic (PC), and electrochemical (EC) method, Fe/TNAs PEC showed the best removal efficiency for methyl orange degradation. Furthermore, the Fe/TNAs PEC system also performed better removal efficiency than that of photolysis method in *E. coli* degradation experiments.

## 1. Introduction

Titanium dioxide (TiO_2_) is one of the most promising photocatalysts because of its excellent properties such as resistance to acid and alkali corrosion, relative low price, non-toxicity, and small environmental footprint. Therefore, it has been widely applied in water treatment [1], atmospheric VOC (volatile organic compounds) removal [2], disinfection [3,4], hydrogen production [5,6,7], CO_2_ reduction [8], DSSC (dye-sensitized solar cell) [9,10], and sensing devices [11], etc. The photo-generated holes can react with water molecular to form hydroxyl radicals, which is a powerful oxidant, to oxidize most organic compounds such as dyes, PPCPs (pharmaceutical and personal care products) [6,12], VOCs [2], and PCDD/Fs (polychlorinated dibenzodioxins and polychlorinated dibenzofurans) [13,14]. On the other hand, the photo-generated electrons can react to generate hydrogen, which is a kind of clean energy. Moreover, the photo-generated electrons can also be applied for environmental applications, e.g., to reduce TCE (trichloroethylene) [15] and to react with ammonia borane (NH_3_BH_3_) to produce hydrogen [16].

The large band gap of 3.2 eV is the main obstacle for TiO_2_ applications. Many researchers employed non-metals such as N [17,18], C [19], F [20], and S [21] to dope into TiO_2_ to reduce the bandgap. The non-metal element can replace the oxygen vacancy to manipulate the lattice of TiO_2_ to form TiO_2−x_A_x_ (where A refers to non-metal elements). The other way is applying metals and/or metal oxides such as Ag [6,22], Pt [23], Au [22,24], Pd [25], Fe [26], and Cu_2_O [5] to form a new Femi level of the as-synthesized metal-modified TiO_2_ to reduce the bandgap. Among various doping elements, trivalent iron is a transition metal that is easy to obtain, low in price, and difficult to react with organic compounds. The activity increase found for some reactions with Fe-doped titania has been attributed to a faster diffusion of reaction intermediates in comparison to pure TiO_2_ [27]. The bandgap of Fe-TiO_2_ was reduced from 3.08 to 2.2 eV after Fe modification and showed effective removal rate of phenol under 700 W visible light [28]. Zafar et al. (2021) used the iron doped titanium dioxide nanotubes (Fe-TNT) photocatalyst in various environmental water matrices including tap water, ultra-pure water, seawater, surface water, and deionized water under visible light. Results showed the Fe-TNT performed significant CR (Congo Red) decolorization, and TOC and COD removal in deionized water [29]. 

The other challenge of TiO_2_ application is the rapid recombination of electron-hole pairs. As the light with suitable energy (i.e., depends on the wavelength) TiO_2_, the electrons can overcome the bandgap to jump from the valence band to the conduction band, leading the holes in the valence band. The photo-generated electrons would recombine with electrons rapidly, therefore, reducing the oxidation and/or reduction ability of TiO_2_. By applying a bias potential between TiO_2_-anode and cathode in the photoelectrochemical system can significantly reduce the recombination of electron-hole pairs [30]. The electron lifetime can increase from 231.78 to 375.59 ms for silver modified TNAs than pure TNAs at +1.0 V (vs. Ag/AgCl) bias potential [6].

Bacteria is one of the indicators of water quality in wastewater treatment plants. To deal with bacteria issue, chlorination is the most used method for wastewater disinfection. However, chlorination disinfection methods cause concerns regarding the toxicity of chlorine residuals and its byproducts [31,32]. UV irradiation has become one of the most important alternatives to chlorination for wastewater disinfection nowadays. Tosa and Hirata (1999) found that the dose of UV light required for 90% and 99% inactivation of EHEC O157:H7 was 1.5 and 3.0 mW scm^−2^ [3]. Further UV disinfection combined with TiO_2_ [4], ozone [33], and radio frequency electric field [34] were also developed.

In this study, ferric nitrate was used as a precursor to modify titanium dioxide to improve the photocatalytic activity of titanium dioxide. The iron modified TNAs are characterized to demonstrate its physical, chemical and photoelectrochemical properties. The *E. coli* was elected as the bio-target for evaluating the PEC oxidation ability of Fe/TNAs for the very first time.

## 2. Materials and Methods

### 2.1. Preparation of Fe Modified Titanium Dioxide Nanotube Arrays (Fe/TNAs)

The titanium foil (99.5%, Zhang Jia Ltd., New Taipei City, Taiwan) was cut into the size of 2 × 2.5 cm and then was washed via acetone, ethanol, and DI water sequent to remove dust and impurities. After cleaning, the titanium foil was dried in the air. The pre-treated titanium foil was used to synthesize TNAs by anodization method. The positive electrode was connected to a titanium sheet, while the negative electrode was connected to a platinum (Pt) electrode in the solution of ammonium fluoride (0.5 wt % NH_4_F (98%, Alfa Aesar, Haverhill, MA, USA) and 6 vt % H_2_O) as the etching solution. The titanium foil was etched at a constant voltage of 40 V for 1 h to obtain TNAs. The as-synthesized TNAs was rinsed with DI water, and dried in oven at 40 °C. To increase the crystalline, the TNAs was calcined at 450 °C for 3 h. The iron deposited TNAs were synthesized by square wave voltammetry electrochemical deposition method (SWVE). First, pH of mixed solution of 0.2 M Fe(NO_3_)_3_·9H_2_O (98%, Alfa Aesar, Haverhill, MA, USA) and 0.05 M NaBH_4_ (99%, Alfa Aesar, Haverhill, MA, USA) was adjusted to 3.5 via 0.5 M HNO_3_ (65%, MERCK, Darmstadt, Germany). The TNAs sample was then immersed in the prepared solution for 20 min. The initial and final applied voltage was set as −1.0 and 0.0 V, respectively, with amplitude of 0.005 V and frequency of 2 Hz for the SWVE method. The as-synthesized samples were named as Fe/TNAs-0.2 and Fe/TNAs-0.5 according to the concentration of Fe(NO_3_)_3_·9H_2_O as 0.2 M and 0.5 M, respectively. The as-synthesized Fe/TNAs was rinsed by DI water and dried at 40 °C for two hours.

### 2.2. Characterization of Nanotube Arrays

The crystal structure of TNAs and Fe/TNAs was investigated by X-ray diffraction (XRD) (X’Pert Pro MRD, PANalytical, Almelo, The Netherlands) using a Cu Kα source at a wavelength of 0.154 nm. JCPDS PDF card database was selected as the identification of XRD peaks. The morphology was studied using a field-emission scanning electron microscope (Nova NanoSEM 430 FEI, Hillsboro, OR, USA). The specific surface area of TNAs and Fe/TNAs were measured by BET (ASAP 2020 N (S/N: 1195), Micromeritics, Norcross, GA, USA) analysis under 740 mmHg of pressure (P_0_) and 77.350 K of bath temperature. Raman analysis was conducted by using 532 nm Laser, with 1800 cm^−1^ grating and 50 s exposure time, operating by Labspec5 software. The X-ray photoelectron spectroscopy (XPS) experiments were conducted on the TNAs and Fe/TNAs using a PHI 5000 Versa Probe system (Physical Electronics, Chanhassen, MN, USA). The binding energy that was obtained from the XPS spectra was calibrated with reference to the C1s peak at 284.8 eV. The UV-vis absorption spectra were measured in diffused reflection mode using an integrating sphere (ISV-922, Jasco, Tokyo, Japan) that was attached to a Jasco V-750 UV-vis DRS spectrometer (V-750, Jasco, Tokyo, Japan).

### 2.3. PEC and Electrochemical Measurements

All PEC experiments were carried out in three-electrode mode at room temperature. A Fe/TNAs (2 cm^2^) and a Pt wire (99.997%, Alfa Aesar, Haverhill, MA, USA) served as a working electrode and a counter electrode, respectively. An Ag/AgCl (3 M KCl, ΩMetrohm, Herisau, Switzerland) electrode was selected as a reference electrode. A self-designed H-type reactor separated the anode and cathode to enhance the pollutants degradation. These two chambers were connected with a cation-exchanged membrane (Nafion 212, DuPount, Wilmington, DE, USA) to keep the ion balance in the system. A quartz window (7 cm^2^) on the side of the anode chamber provided high optical quality. For the photocurrent measurement, the operating light source is a 100 W mercury lamp (GGZ100, Shanghai Jiguang, Shanghai, China) at a bias potential of +1.0 V (vs. Ag/AgCl) in the electrolyte of 0.1 M NaCl (99%, Avantor, Radnor, PA, USA), while a platinum (Pt) and Ag/AgCl electrode work as the counter and reference electrode, respectively. Electrochemical impedance spectroscopy (EIS) was performed under an open circuit voltage with frequencies in the range from 10 kHz to 10 mHz with an AC voltage with an amplitude of 5 mV. The potential for I-V, I-t curve measurements and PEC degradation experiments were controlled by an electricity workstation (ΩMetrohm-Autolab B.V. PGSTAT204, Herisau, Switzerland). For the methyl orange (85%, Sigma Aldrich, St. Louis, Missouri, USA) degradation, the PEC, photocatalytic (PC), electrochemical (EC) and photolysis (P) experiments were examined at the illumination using 100 W Hg lamp. Applied voltage in PEC and EC process was +1.0 V (vs. Ag/AgCl). *E. coli* is a gram-negative bacteria and used as an indicator of fecal pollution in water. *E. coli* (CCRC 10674) was purchased from the Bioresource Collection and Research Center of the Food Industry Research and Development Institute (Hsinchu City, Taiwan). *E. coli* K-12 strain CCRC 10674 was grown aerobically in 100 mL of brain–heart infusion at 37 °C for 18 h and the micro-organisms were cultured at 25 °C. Consequently, 1 mL aqueous samples containing 70 CFU/mL initial *E. coli* concentration were prepared and subjected to treatment. In this study, *E. coli* was used to evaluate the Fe/TNAs PEC degradation ability. The operated light source is 100 W Hg lamp at a bias potential of +1.0 V (vs. Ag/AgCl), while a reference electrode of Ag/AgCl, counter electrode of Pt wire, and a working electrode of Fe/TNAs-0.5 were employed.

## 3. Results and Discussions

### 3.1. Surface Morphology of TNAs and Fe/TNAs

**Figure 1:**
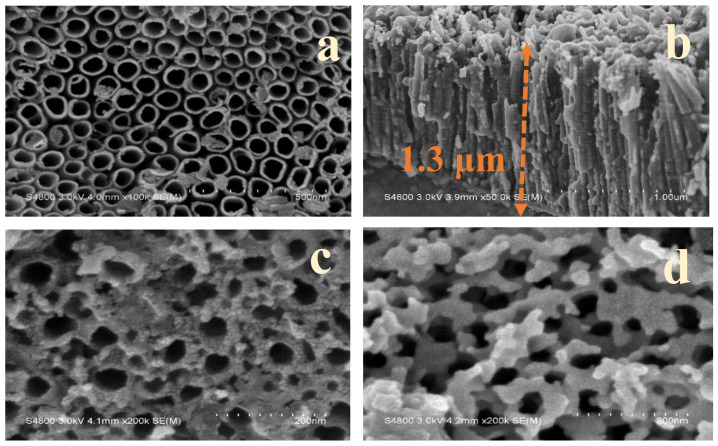
SEM images for (**a**) TNAs (×100 k), (**b**) cross-section image of TNAs (×50 k), (**c**) Fe/TNAs-0.2 (×200 k), and (**d**) Fe/TNAs-0.5 (×200 k) (parentheses shows the magnification).

In this study, SEM was used to identify the morphology of the surface of the material, and to explore the tubular structure of TNAs and Fe/TNAs prepared by the anodic oxidation etching method. Figure 1 shows the SEM images of TNAs and Fe/TNAs, respectively. Figure 1a shows an array of titanium dioxide nanotubes synthesized by anodizing method. The structure is compact and neatly arranged, and the diameter of the tube were approximately in the range of 50–100 nm. Many previous studies have reported a linear correlation between the diameter of titanate nanotube arrays and the applied anode voltage (Marck et al., 2017; Yasuda et al., 2007). Figure 1b shows the cross-sectional view of the un-modified titanium dioxide nanotube arrays with a length about 1.3 μm. Cao et al. (2016) applied anodization method to synthesize titanium dioxide nanotube arrays and found that the length of TNAs increases with the time of anodization [35]. Figure 1c,d shows the iron modified titanate nanotube arrays synthesized (Fe/TNAs) via SWVE method with 0.2 and 0.5 M Fe(NO_3_)_3_·9H_2_O precursor, respectively. The structure of the nanotube is not damaged after ferric nitrate modification, and the iron is successfully deposited on the surface of the TNAs in the form of sol particles. The morphology of iron is irregular, and it is evenly distributed and accumulated around the surface of TNAs. Notably, as the concentration of precursor of iron nitrate increases, the iron deposits increased on the TNAs. Previous literature showed a similar deposition morphology, confirming that sodium borohydride can successfully reduce Fe^3+^ in ferric nitrate to zero-valent iron Fe^0^ [36]. Table 1 shows the results of EDX analyses of TNAs, Fe/TNAs-0.2, and Fe/TNAs-0.5, respectively. The measured elements include C, O, Ti, and Fe, individually. The atomic percentage of Fe were 0.00, 0.93, and 7.44% for TNAs, Fe/TNAs-0.2, and Fe/TNAs-0.5, respectively, illustrating that the Fe were successfully deposited on the surface of TNAs. Notably, as shown in Figure 1d, most surface of the TNAs were covered by Fe, therefore, the Ti content is much less in Fe-TNAs-0.5 (16.51%) than that of TNAs (34.15%) and Fe/TNAs-0.2 (33.84%). Figure S1 shows the BET nitrogen adsorption-desorption isotherms of TNAs and Fe/TNAs. The measured surface area of TNAs and Fe/TNAs were 0.1464 and 0.0029 m^2^/g, respectively.

### 3.2. XRD Analyses

**Figure 2:**
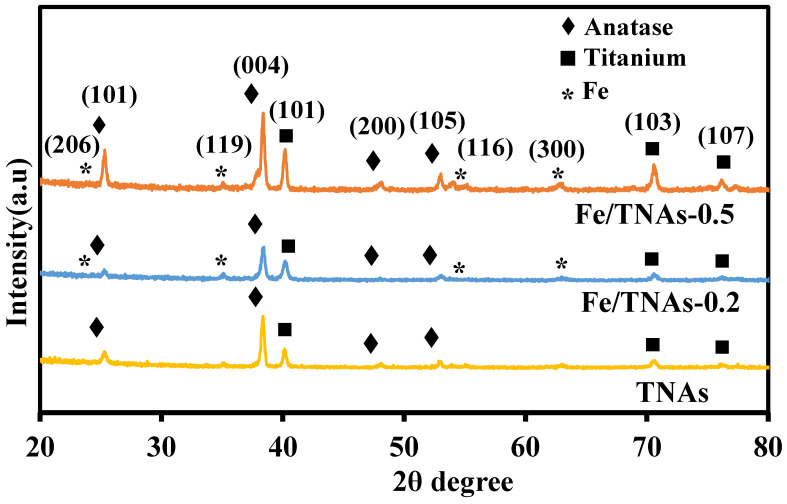
The XRD patterns for TNAs, Fe/TNAs-0.2, and Fe/TNAs-0.5.

Figure 2 shows the XRD results for TNAs, Fe/TNAs-0.2, and Fe/TNAs-0.5. Compared with the standard chart of the Joint Committee for Powder Diffraction Files (JCPDFs: 21–1272), the XRD results show that TNAs exhibits anatase characteristic peaks at 2θ = 25.3°, 37.8° and 54.0°, corresponding to the crystal planes of (101), (004), and (105), respectively. No rutile phase was observed due to the insufficient temperature [5,15]. Notably, Fe/TNAs-0.2 and Fe/TNAs-0.5 show the diffraction peaks of Fe at 2θ = 24.16°, 35.74°, 54.23°, 62.26°, corresponding to the crystal planes of (012), (110), (116), and (214) (JCPDS file number: 00-001-1053), respectively, indicating that the Fe nanoparticles have been deposited on the surface of TNAs. Similar results of XRD analyses for TiO_2_-Fe_2_O_3_ were found in literature, showing that the successfully deposited iron [37]. Moreover, with the increase amount of Fe (see Table 1), the diffraction peak intensities corresponding to Fe increases as well. Figure S2 shows the Raman analyses for bare TNAs and Fe/TNAs. In general, anatase phase of titanium dioxide has six Raman active modes 1A_1g_, 2B_1g_, and 3E_g_. TNAs and Fe/TNAs show solo peak at 143.1 and 144.5 cm^−1^, respectively, that corresponding to the active mode of E_g_ of anatase phase of TiO_2_. The results in Ramana analyses are in good agreement with the XRD observations. Notably, the main E_g_ mode located at around 144 cm^−1^ broadened and shifted toward higher wavenumber in Fe/TNA (inset plot in Figure S1), indicating that the Fe^3+^ are successfully incorporated into the TiO_2_ framework, replacing Ti^4+^ cations. This results in in consistent to the findings of Fe-TiO_2_ NPs [38].

### 3.3. XPS Analyses

**Figure 3:**
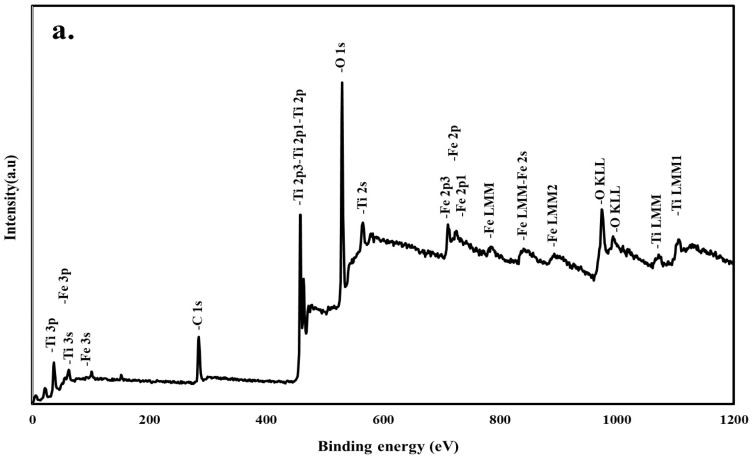

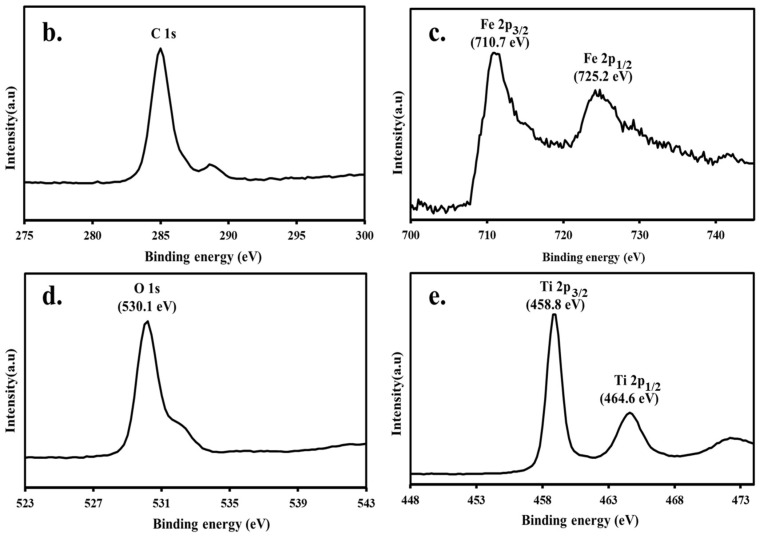
XPS spectra of (**a**) XPS spectrum of Fe/TNAs-0.5, (**b**) C 1s, (**c**) Fe 2p, (**d**) O 1s, and (**e**) Ti 2p.

The electron energy spectrometer (XPS), as shown in Figure 3a–e, are used to clarify the composition of elements of Fe/TNAs and to analyze the energy spectrum. It is also used to identify the uniformity and dispersion of surface elements of materials. Figure 3a is the full-energy spectra of Fe/TNAs-0.5, showing that C, O, Ti, and Fe are distributed on the surface of the material. Figure 3b shows the characteristic peak of C 1s at 284.5 eV. The C element was attributed to the addition to the apparatus during the analysis. Figure 3c shows the characteristic peaks of Fe at 710 eV and 725 eV, corresponding to the element states of 2p_3/2_ and 2p_1/2_, respectively. Notably, the enhancement in binding energy in the elemental state of 2p_3/2_ is due to the fact that Fe^3+^ disperses to Ti^4+^ in the anatase lattice and forms Fe−O−Ti bonds [39]. Zue et al. (2006) used sol-gel method to synthesize Fe-TiO_2_ and indicated that the binding energies are 711.0–711.8 eV and 725.4–726.0 eV for 2p_3/2_ and 2p_1/2_, respectively, of Fe^3+^ [39], while Huerta-Flores, et al., (2021) illustrated that two main peaks were identified around 710.7 eV and 724.8 eV attributed to Fe 2*p*_3/2_ and 2*p*_1/2_ levels of Fe^3+^ in Fe_2_O_3_ material [40]. Above findings are different to the observations of Fe^0^ that showing the characteristic peaks at 707.8 and 720.2eV for Fe 2*p*_3/2_ and 2*p*_1/2_, respectively, in Wang et al. (2018) [41]. Hsieh et al. (2010) also indicated that two major peaks at 707.0 and 719.1 eV are representing the Fe 2*p*_3/2_ and 2*p*_1/2_, corresponding to the zero-valent iron [42]. Hence, we confirmed the state of Fe in Fe/TNAs is Fe^3+^ instead of Fe^0^ as the findings in Figure 3c. Figure 3d shows that O has a characteristic peak at 530 eV, which represents the formation of Ti or Fe oxides [43]. Figure 3e shows that the characteristic peaks of Ti at 458 eV and 464 eV correspond to Ti 2p_3/2_ and Ti 2p_1/2_ respectively, confirming that Ti exists in the form of Ti^4+^ [44]. Table 2 shows the element percentage of Fe/TNAs-0.2 and Fe/TNAs-0.5 by XPS analysis. The Fe content for Fe/TNAs-0.2 and Fe/TNAs-0.5 is 0.9% and 2.9%, respectively, that are less than the results of EDX analyses. This is because the distribution of iron particles on the surface of the titanium sheet is uneven, and the particle size is different. The comparison of element components showed a slight difference between EDX and XPS because of the following reasons: (a) the range of analytical depth for XPS and EDX is within 10 and 100–1000 nm, respectively; (b) the analytical area of samples was larger in XPS than that in EDX; (c) the appearance of the C element can be attributed to extrinsic carbon during the sample fabrication and/or the XPS instrument itself.

### 3.4. UV-Vis DRS Analyses

The absorption coefficient of the thin films was calculated with the following formula:

(1) $$\alpha \left(hv\right)=-\frac{1}{d}\ln\left(\frac{T}{{\left(1-R\right)}^{2}}\right)$$

where *α* is the absorption coefficient, *d* is the thickness of the film, and for a particular wavelength *T* is transmission and *R* is the reflectance characteristics of the film.

(2) A(hv−EG)=(αhv)1n

**Figure 4:**
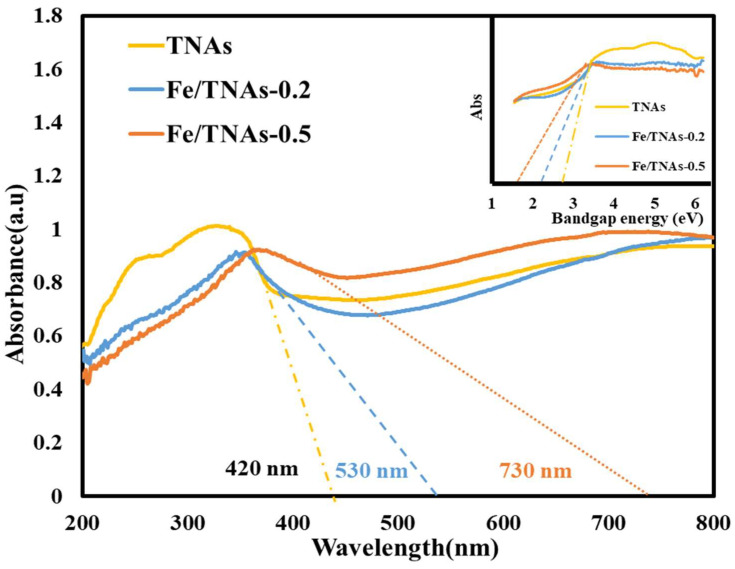
UV-vis DRS spectra of TNAs, Fe/TNAs-0.2, and Fe/TNAs-0.5; insert plot: bandgap energy for TNAs, Fe/TNAs-0.2, and Fe/TNAs-0.5.

The optical band gap is calculated by plotting (*αhv*)^1/*n*^ versus *hv* (i.e., eV)*,* where α is the absorption coefficient of the material, *h* is the Planck constant, *ν* is the photon’s frequency, *A* is a constant, and *E_G_* is the bandgap energy. As to the constant *n* is 0.5 and 1 for indirect and direct band gap materials, respectively. TiO_2_ has an indirect band gap [38]. As shown in Figure 4, the absorbance wavelength is 420, 530, 730 nm for TNAs, Fe/TNAs-0.2 and Fe/TNAs-0.5, respectively. The absorbance wavelength of TNAs red shift to longer wavelength region after Fe nanoparticles modification illustrating the better light utilization. Band gap (*E*_G_) can be calculated according to the Tauc formula [45] as shown Equation (2) above. The inset plot in Figure 4 shows the corresponding bandgap is 2.9, 2.3, and 1.7 eV for TNAs, Fe/TNAs-0.2 and Fe/TNAs-0.5, individually. The results here are similar to the findings of Fe-TiO_2_ thin film [46] and Fe-TiO_2_ nanoparticles [38,47] that applying Tauc formula to investigate the bandgap energy. In general, there is a good correlation between light absorption and photocatalytic activity. Stronger light absorption correlated with higher photocatalytic activity. UV-vis DRS spectra results confirmed that Fe nanoparticles were successfully deposited on TNAs.

### 3.5. Photocurrent Measurement

**Figure 5:**
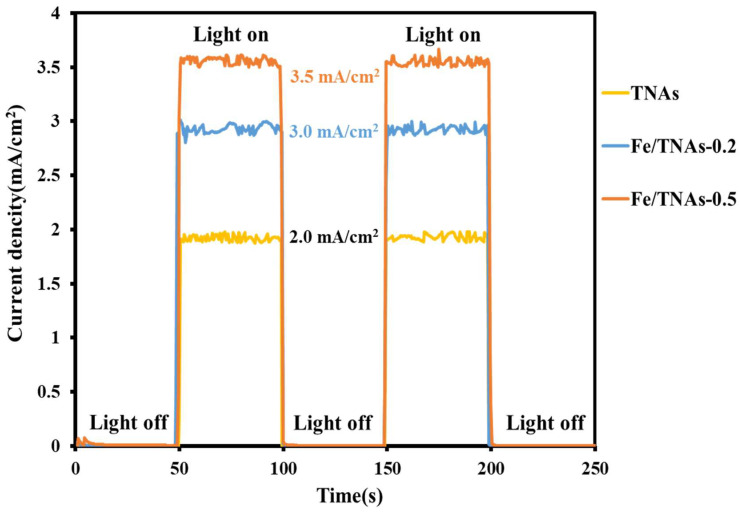
Photocurrent-time response of TNAs, Fe/TNAs-0.2, and Fe/TNAs-0.5 (light source: 100 W hg lamp, applied potential: +1.0 V (vs. Ag/AgCl), electrolyte: 0.1 M NaCl, reference electrode: Ag/AgCl, counter electrode: Pt wire, and working electrode: TNAs, Fe/TNAs-0.2, and Fe/TNAs-0.5).

Figure 5 shows the measured photocurrent diagram by using TNAs, Fe/TNAs-0.2 and Fe/TNAs-0.5 as the photo-anode in a PEC system. As shown in the figure, the photocurrent was monitored under a 50 s on-off cycle. The photocurrent of three tested photo-anodes is about 1–4 μA when the illumination is off. Because there is no light to excite the photoelectrons on the surface of the material, leading to no photocurrent is generated. When the light is on, all materials generate photocurrent as a stable straight line, which means that the photoelectrochemical performance of the photo-anodes tend to a stable state. The photocurrent densities of TNAs, Fe/TNAs-0.2, and Fe/TNAs-0.5 are 2.0, 3.0, and 3.5 mA/cm^2^, respectively. Notably, the photocurrent was 1.75 times higher for Fe/TNAs-0.5 than that of pure TNAs at the same illumination. TNAs modified by iron can excite more photo-generated electrons than TNAs under irradiation, which promotes the increase of photocurrent, indicating that more electron-hole pairs are generated on the surface of the material, resulting to better degrade pollutants.

### 3.6. Electrochemical Impedance Spectrum

**Figure 6:**
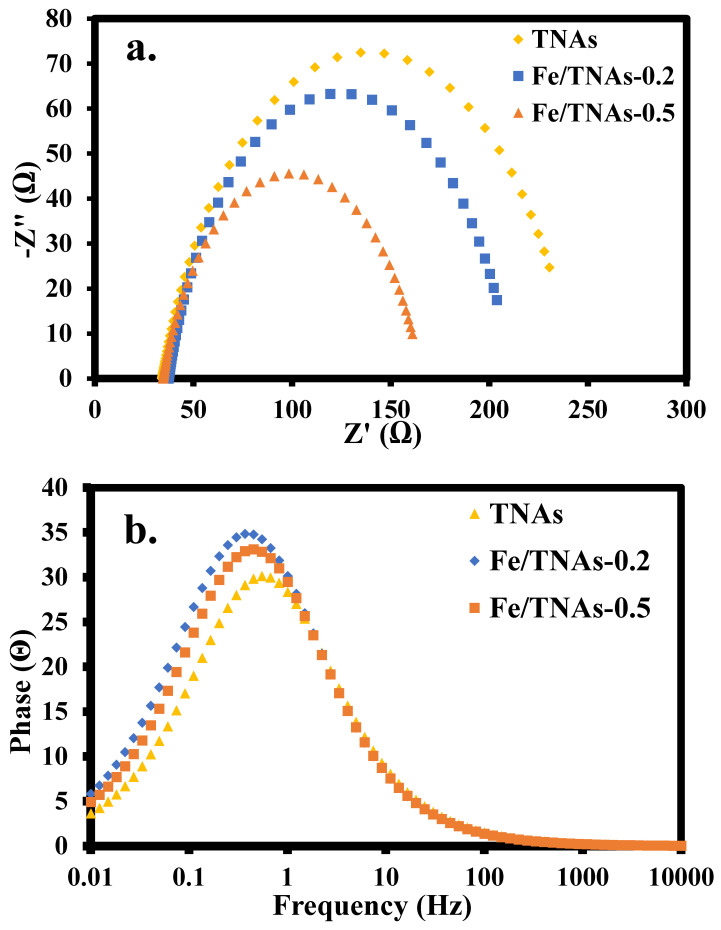
Nyquist plot (**a**) and Bode plot (**b**) based on EIS analyses of TNA, Fe/TNAs-0.2, and Fe/TNAs-0.5.

In this study, electrochemical impedance spectrum (EIS) was used to analyze the electron-hole separation ability and electron transfer characteristics of TNAs, Fe/TNAs-0.2 and Fe/TNAs-0.5 under light irradiation. The smaller the diameter of the arc refers to the better the separation of electrons and holes, resulting in better photoelectrochemical ability. Figure 6a shows the Nyquist plots of TNAs, Fe/TNAs-0.2, and Fe/TNAs-0.5 by EIS analysis, indicating that the diameter of each semicircle follows the order: TNAs > Fe/TNAs-0.2 > Fe/TNAs-0.5, which demonstrating that the electron mobility of TNAs was enhanced by Fe modification. Figure 6b shows the Bode plot obtained by EIS analysis for three photo-anodes. The maximum frequency obtained from the figure is then fitted with the equation of $\tau_{el}=\frac{1}{2\pi \times f_{max}}$ to calculate the electron lifetime. Table 3 shows the electron lifetime was 290.3, 354.7, and 433.3 ms, corresponding to the *f_max_* of 0.548, 0.449, and 0.367 Hz in Figure 6a, for TNA, Fe/TNAs-0.2, and Fe/TNAs-0.5, respectively. Table 3 summarizes the result of fitted equivalent circuit for TNA, Fe/TNAs-0.2, and Fe/TNAs-0.5, respectively. Notably, R_s_ refers to the resistance of the solution and R_p_ represents the charge transfer at the interface between Fe/TNAs and electrolyte, while the CPE refers to the constant phase element [5]. The R_p_ was 290.5, 143.33, and 130.97 for TNA, Fe/TNAs-0.2, and Fe/TNAs-0.5, individually, with +1.0 V (vs. Ag/AgCl) of bias potential. According to the results of Figure 6 and Table 3, we can preliminarily conclude that TNAs modified with iron can effectively reduce the recombination of electron holes, prolong the residence time of electrons or increase the mobility of electrons in the PEC system.

### 3.7. Mott-Schottky Plot for TNAs and Fe/TNAs

The Mott-Schottky plot is performed by applying the electrochemical impedance method, which is carried out in the range of −0.6 V to +0.2 V (vs. Ag/AgCl) under frequency of 100 Hz and amplitude of 10 mV. The type and flat band potential of semiconductor were determined by following equation [40,48,49]:

(3) $$\frac{1}{C^{2}}=\frac{2}{\varepsilon \varepsilon_{0}A^{2}qN_{D}}\left(V-V_{fb}-\frac{KT}{q}\right)$$

where ε is the dielectric constant of TiO_2_ (i.e., 31 for anatase) [50,51], ε_0_ is the permittivity of vacuum (8.85 × 10^−14^ F cm^−1^), *A* is the area of photo-anode (4 cm^2^), *q* is the elementary charge (1.6 × 10^−19^ C), *N_D_* is the density of dopants (cm^−3^), *V* is the applied voltage, *V_fb_* is the flat band potential, *K* is the Boltzman constant (1.381 × 10^−23^ J K^−1^), and *T* is the absolute temperature (298 K). In addition, the charge carrier density (*N_D_*) of semiconductor was also calculated from the slope of Mott–Schottky plots, which is following equation [48,52,53]:

(4) $$\frac{dC^{-2}}{dV}=\frac{2}{q\varepsilon \varepsilon_{0}N_{D}A^{2}}$$

**Figure 7:**
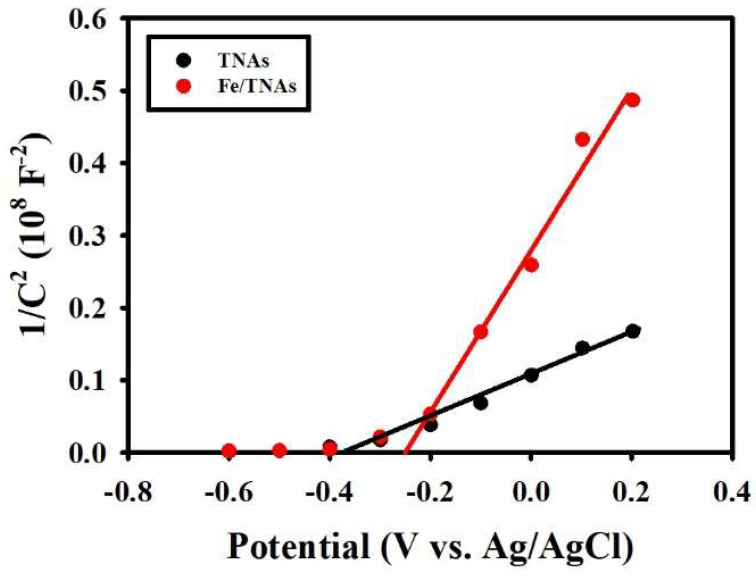
Mott-Schottky plot of TNAs and Fe/TNAs at frequency of 100 Hz (working electrode: TNAs (or Fe/TNAs); counter electrode: Pt wire; reference electrode: Ag/AgCl; temperature: 298 K; electrolyte: 0.1 M NaCl; light source: 100 W Hg lamp; amplitude: 10 mV).

As shown in Figure 7, both TNAs and Fe/TNAs show the positive slope, confirming that Fe depositing on TNAs does not change its n-type semiconductor property. Moreover, the *V_fb_* of TNAs and Fe/TNAs was −0.34 and −0.25 V (vs. Ag/AgCl), individually, which were estimated from the intercept with the X-axis on the linear plot. The positive shift of *V_fb_* in Fe/TNAs photo-anode implies the facilitating of photo-generated electron-hole pairs separation and transfer, which is corresponding to EIS results (Figure 6) [48]. The charge carrier density (*N_D_*) of TNAs (9.04 × 10^21^) was higher than that of Fe/TNAs (2.52 × 10^21^ cm^−3^). This was attributed to the slope of Fe/TNAs photo-anode (1.13 × 10^8^) which was higher than that of TNAs photo-anode (3.15 × 10^7^), indicating the significant decrease of charge carrier density of Fe/TNAs. Similar observations were found in Freitas et al. (2014), indicating that the increasing of charge carrier density was attributed to a high level of defects caused by oxygen vacancies [54].

In addition, when photo-anode is contacted with the electrolyte, the electrons on photo-anode will transfer to electrolyte spontaneously to form depletion region with positive charge between the interface of photo-anode and electrolyte. The width of depletion region (*W*) was also derived from the Mott–Schottky plot relationship and is described by following equation [48,54]:

(5) $$W={\left(\frac{2\varepsilon \varepsilon_{0}\left(V-V_{fb}\right)}{qN_{D}}\right)}^{\frac{1}{2}}$$

The depletion region of TNAs and Fe/TNAs was 0.311 and 0.661 nm, individually. As the result, the oxygen vacancies were decreased with the substitution of Ti^4+^ by Fe^3+^ at the surface, enlarging the space charge region at TiO_2_-electrolyte interface [48]. Furthermore, the conduction band (*E_CB_*) is determined from the following equation for n-type semiconductor:

(6) $$E_{CB}=V_{fb}+KT\;\ln\left(\frac{N_{D}}{N_{c}}\right)$$

where *K* is the Bolzman constant (8.617 × 10^−5^ eV K^−1^) and *N_c_* is the effective density of states (DOS) at the conduction band edge, which can be calculated by using equation: $N_{c}=2{\left(\frac{2\pi \;m_{eff}KT}{h^{2}}\right)}^{\frac{3}{2}}$. The *m_eff_* of 9 *m*_0_ (for TiO_2_ anatase) is used for *N_c_* calculations [55], where *m*_0_ is the mass of electron (9.109 × 10^−31^ kg), *K* is the Bolzman constant (1.381 × 10^−23^ J K^−1^), *T* is the absolute temperature (298 K), and *h* is the Planck constant (6.626 × 10^−34^ J s). By applying the equation, *N_c_* is determined as 6.71 × 10^20^ cm^−3^. Therefore, the conduction band −0.27 and −0.22 V for TNAs and Fe/TNAs, respectively. Furthermore, UV-vis DRS results showed that the band gap of TNAs and Fe/TNAs was 2.9 and 1.7 eV, respectively, thus, the position of the valence band was at approximately 2.63 and 1.48 V for TNAs and Fe/TNAs, respectively.

### 3.8. Methyl Orange Removal

**Figure 8:**
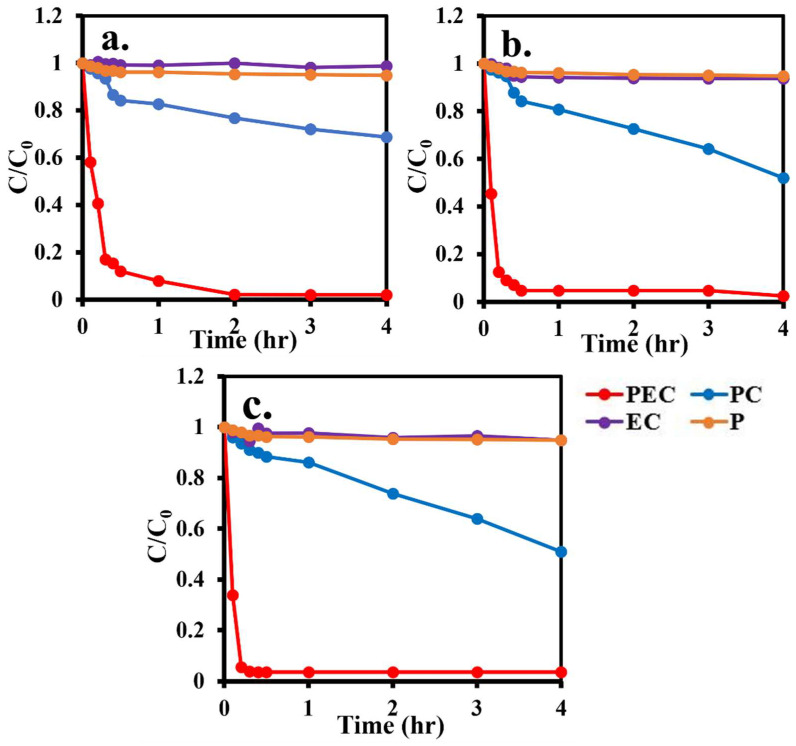
Comparison of methyl orange degradation in PEC, photocatalytic (PC), electrochemical (EC) and photolysis (p) processes by (**a**)TNAs, (**b**) Fe/TNAs-0.2, and (**c**) Fe/TNAs-0.5 (light source: 100 W hg lamp, applied potential: +1.0 V (vs. Ag/AgCl), electrolyte: 10 ppm methyl orange in 0.1 M NaCl solution, reference electrode: Ag/AgCl, counter electrode: Pt wire, and working electrode: TNAs, Fe/TNAs-0.2, and Fe/TNAs-0.5).

Four methyl orange removal processes, namely, PEC, PC, EC and P processes were conducted to evaluate the degradation efficiency of TNA, Fe/TNAs-0.2, and Fe/TNAs-0.5. As shown in Figure 8, PEC process was the most efficient way to degrade MO among three methods studied. The complete removal of 10 ppm of MO was observed after 120, 50 and 30 min for the PEC method by using TNA, Fe/TNAs-0.2, and Fe/TNAs-0.5, respectively. The order of MO degradation by TNA, Fe/TNAs-0.2, and Fe/TNAs-0.5 is in consistent to the photocurrent densities of TNAs, Fe/TNAs-0.2, and Fe/TNAs-0.5 that are 2.0, 3.0, and 3.5 mA/cm^2^, respectively. According to the EIS analyses, the electron lifetime was 290.3, 354.7, and 433.3 ms for TNA, Fe/TNAs-0.2, and Fe/TNAs-0.5, respectively, illustrating that the freedom of electrons has increased. This enhanced PEC performance was attributed to the Fe modified as evidence of results of EDX and XPS.

Briefly, the MO degradation efficiency in four methods follows: PEC > PC > P > EC. It was observed that the PEC method provided the most powerful way to degrade MO due to the combination of electrochemical oxidation and photocatalysis [5].

### 3.9. E. coli Removal by Fe/TNAs PEC System

**Figure 9:**
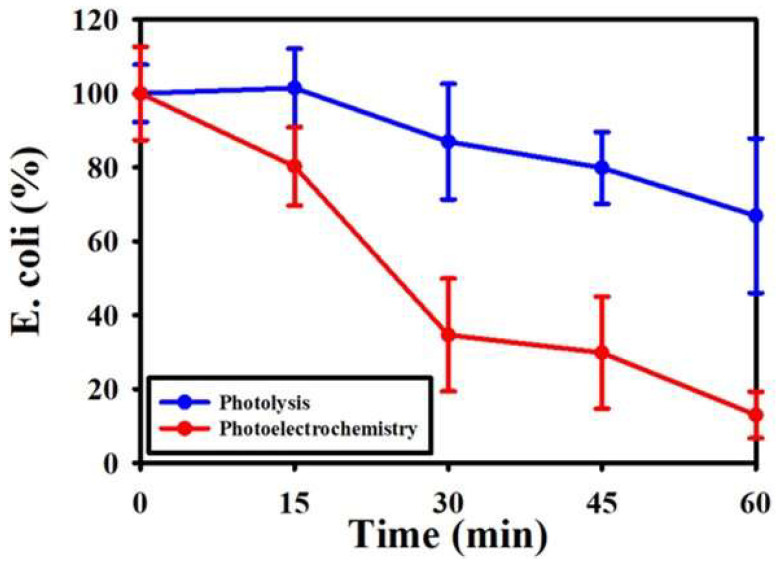
PEC and P removal of *E. coli* (light source: 100 W hg lamp, applied potential: +1.0 V (vs. Ag/AgCl), electrolyte: *E. coli* in 0.1 M NaCl solution, reference electrode: Ag/AgCl, counter electrode: Pt wire, and working electrode: Fe/TNAs-0.5).

*E. coli* was selected as the bio-target for investigating the PEC disinfection ability. Figure 8 shows the *E. coli* removal efficiency was 87.02% by using Fe/TNAs-0.5 as the photo-anode in the PEC system within the reaction time of 60 min. *E. coli* degradation by photolysis, the same light source (100 W Hg lamp) in the PEC, was conducted for comparison. The images of *E. coli* removal by TNAs and Fe/TNAs PEC system were demonstrated in Figure S3. As shown in Figure 9, only 33.1% of *E. coli* was removed in the photolysis procedure. It is well known that UV light is commonly used in traditional water treatment system. According to the literature, inactivation of EHEC O26 with photoreactivation is 2.2 times greater than that without photoreactivation [3]. In addition, with the addition of TiO_2_ in UV disinfection system, the damage of outer membrane was observed for the cells. TiO_2_ alone can break down lipopolysaccharide (LPS), the outermost layer of the *E. coli* cells [4]. The hydroxyl radicals, which is a kind of most strong oxidants, were responsible for *E. coli* removal in the TNAs PEC system [6]. Compared to photolysis at the same irradiation, PEC system can generate strong oxidants, i.e., hydroxyl radicals [5,6], to degrade *E. coli*. On the other hand, the recombination of electron-hole pairs is greatly reduced in PEC method as evidence of enhanced electron lifetime (see Table 3), that allowed more photo-generated holes to react with H_2_O to form OH⋅ to removal *E. coli*. The result is in consistent to the findings of Ma et al. (2021) that Z-scheme g-C_3_N_4_/TNAs reduced recombination of photo-generated electron-hole pairs [56].

## 4. Conclusions

Fe/TNAs were fabricated by the SWVE method to remove the *E. coli* for the very first time in this study. The SEM results showed that iron NPs were deposited on the surface of the TNAs in the form of sol particles and the tubular structure was not damaged after iron modification. The Fe content on the surface of TNAs was increased with the increasing of the precursor concentration, which demonstrated by EDX and XPS results. XPS also revealed that the binding energy of Fe located at 710 and 725 eV, which represent +3 state of Fe. XRD analyses showed the characteristic peak of Fe at 2θ = 24.16°, 35.74°, 54.23°, and 62.26°, corresponding to the crystal planes of (012), (110), (116), and (214), illustrating the successful loading of iron NPs. Moreover, UV-vis DRS showed that the light absorbance was increased and the bandgap energy was decreased after iron modification to enhance the light utilization. The results of photoelectrochemical performance indicated that Fe/TNAs-0.5 performed the best photocurrent density (3.5 mA/cm^2^) as well as the electron lifetime of Fe/TNAs-0.5 is 1.49 times greater than that of pure TNAs through the EIS and Bode plot analyses, illustrating that the loading of Fe NPs is advantage to the separation of electron-hole pairs to enhance the photoelectrochemical performance. The deep and shallow trap states in bandgap of TNAs play an essential role in the electron mobility and PEC performances. Therefore, investigation of the effect of the traps of the TNAs is recommended for future study. Compared to the P, PC, and EC methods, PEC was the best method for MO degradation by using Fe/TNAs-0.5 as the photo-anode. Furthermore, the Fe/TNAs-0.5 PEC system also performed better removal efficiency than the P method for *E. coli* degradation. To sum up, the iron NPs deposited TNAs were successfully synthesized in this study. Depositing Fe NPs on TNAs not only reduces the bandgap energy of the TNAs but also decreases the electron-hole recombination, therefore, leading to the enhancement of the PEC performances in MO and *E. coli* removal.

## Figures and Tables

**Table 1:** EDX analyses for TNA, Fe/TNAs-0.2, and Fe/TNAs-0.5.

	Elements	C (Atomict %)	O (Atomict %)	Ti (Atomict %)	Fe (Atomict %)	Total
Sample						
TNAs		2.03	63.82	34.15	0.00	100.0
Fe/TNAs-0.2		1.97	63.25	33.84	0.93	100.0
Fe/TNAs-0.5		9.96	66.08	16.51	7.44	100.0

**Table 2:** Element composition of XPS analyses for TNA, Fe/TNAs-0.2, and Fe/TNAs-0.5.

	Elements	C (Atomict %)	O (Atomict %)	Ti (Atomict %)	Fe (Atomict %)
Sample					
Fe/TNAs-0.2		50.9	45.1	3	0.9
Fe/TNAs-0.5		53.4	27.7	16	2.9

**Table 3:** Fitting results of equivalent circuits of TNAs and Fe/TNAs.

	Rs(Ω)	Rp(Ω)	CPE(Ω)	f_maz_	τ_t_ (ms)
TNAs	38.93	209.50	0.0057	0.5484	290.3
Fe/TNAs-0.2	35.68	143.33	0.0066	0.4489	354.7
Fe/TNAs-0.5	34.56	130.97	0.0056	0.3674	433.3

## Data Availability

Data is contained within article.

## Supplementary Materials

The following are available online at https://www.mdpi.com/article/10.3390/nano11081944/s1, Figure S1: BET analysis of (a) TNAs and (b) Fe/TNAs, Figure S2: Raman spectrum of TNAs and Fe/TNAs, Figure S3: Images of *E. coli* removal in photolysis and PEC system.
