# Supplementary material for: Iron Modified Titanate Nanotube Arrays for Photoelectrochemical Removal of E. coli
Source: Nanomaterials (Basel). 2021 Jul 28;11(8):1944. doi: 10.3390/nano11081944 (PMC8398541; doi:10.3390/nano11081944)
Supplement: Supplementary file 1 [file nanomaterials-11-01944-s001.zip › nanomaterials-1298891-SI.pdf]

Iron Modified Titanate Nanotube Arrays for Photoelectrochemical Removal of *E. Coli*

Chia-Hung Chen [1], Yen-Ping Peng [1,*], Ming-Hsun Lin [2], Ken-Lin Chang [1], Yung-Chang Lin [3,4,*] and Jian Sun [5]

Supplementary Materials

Including three figures

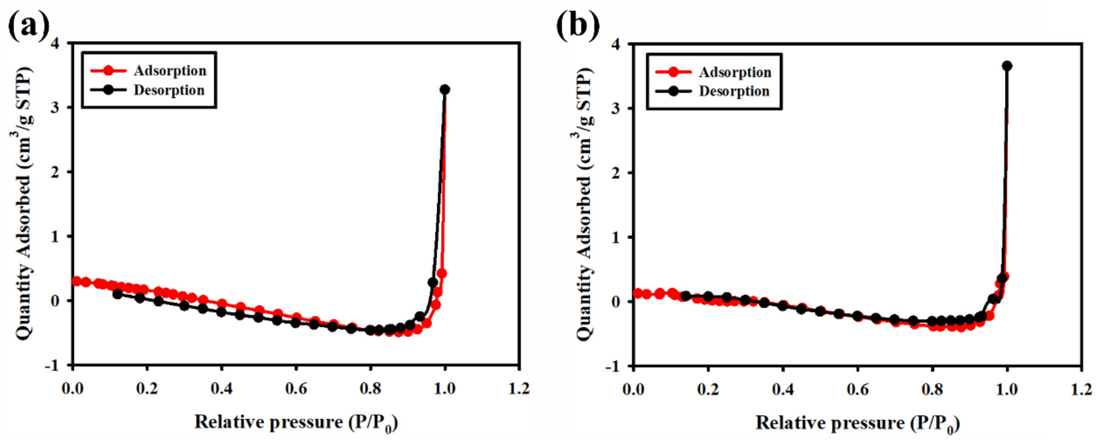

Figure S1. BET analysis of (a) TNAs and (b) Fe/TNAs.

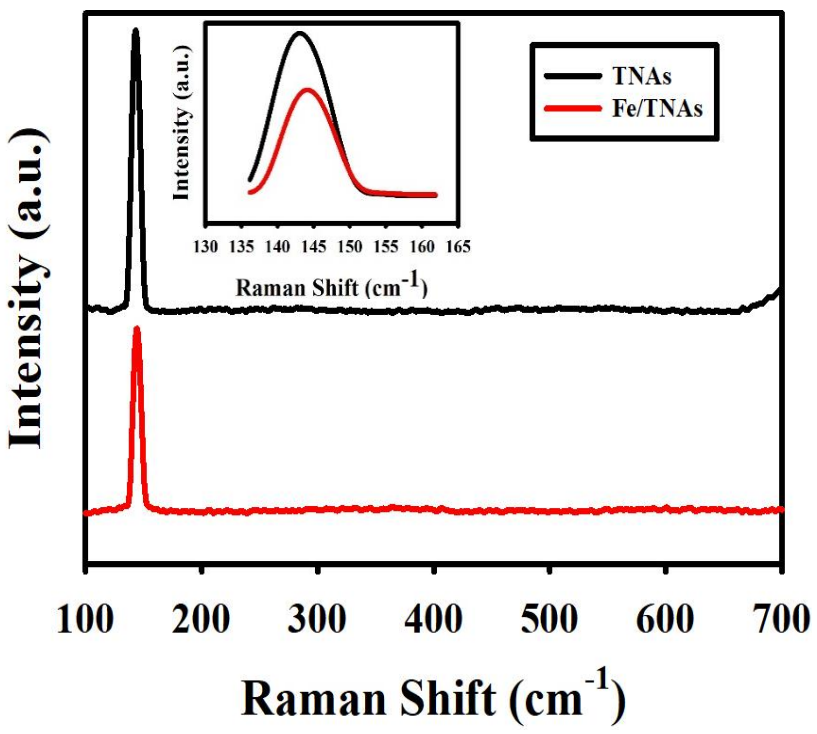

Figure S2. Raman spectrum of TNAs and Fe/TNAs

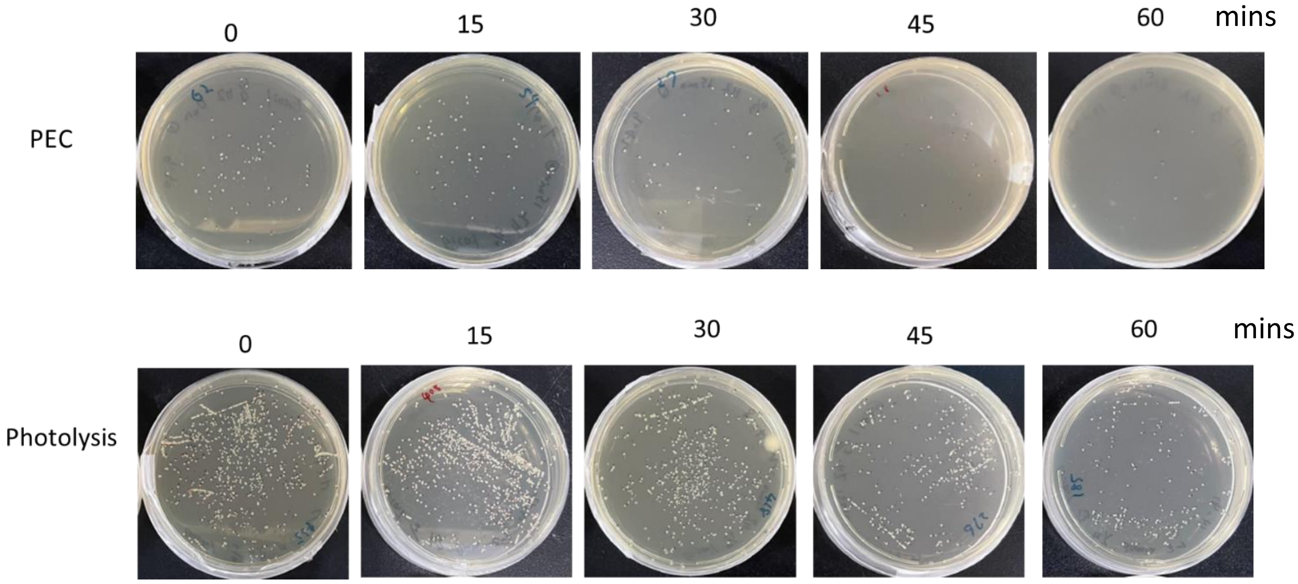

Figure S3. Images of E Coli removal in photolysis and PEC system
